# Examining the effect of evaluation sample size on the sensitivity and specificity of COVID-19 diagnostic tests in practice: a simulation study

**DOI:** 10.1186/s41512-021-00116-4

**Published:** 2022-04-25

**Authors:** Camilla Sammut-Powell, Charles Reynard, Joy Allen, John McDermott, Julian Braybrook, Rosa Parisi, Daniel Lasserson, Richard Body, on behalf of the CONDOR steering committee, Richard Body, Gail Hayward, Joy Allen, Julian Braybrook, Peter Buckle, Paul Dark, Kerrie Davis, Eloise Cook, Adam Gordon, Anna Halstead, Dan Lasserson, Andrew Lewington, Brian Nicholson, Rafael Perera-Salazar, John Simpson, Philip Turner, Graham Prestwich, Charles Reynard, Beverley Riley, Valerie Tate, Mark Wilcox

**Affiliations:** 1 Division of Informatics, Imaging and Data Science, School of Health Sciences, University of Manchester, Manchester, UK; 2 Division of Cardiovascular Sciences, University of Manchester, Manchester, UK; 3 Emergency Department, Manchester Royal Infirmary, Manchester University NHS Foundation Trust, Oxford Road, Manchester, M13 9WL UK; 4 NIHR Newcastle In Vitro Diagnostics Co-operative, Translational and Clinical Research Institute, Newcastle University, Newcastle, UK; 5 Newcastle upon Tyne Hospitals NHS Foundation Trust, Newcastle, UK; 6 Department of Genetics, Manchester University NHS Foundation Trust, Manchester, UK; 7 National Measurement Laboratory, LGC, Queens Road, Teddington, Middlesex, TW11 0LY UK; 8 Warwick Medical School, University of Warwick, Coventry, UK; 9 Department of Geratology, Oxford University Hospitals NHS Foundation Trust, Oxford, UK

**Keywords:** COVID-19, Diagnostic accuracy, Research methodology, Statistical study design, Sample size

## Abstract

**Background:**

In response to the global COVID-19 pandemic, many in vitro diagnostic (IVD) tests for SARS-CoV-2 have been developed. Given the urgent clinical demand, researchers must balance the desire for precise estimates of sensitivity and specificity against the need for rapid implementation. To complement estimates of precision used for sample size calculations, we aimed to estimate the probability that an IVD will fail to perform to expected standards after implementation, following clinical studies with varying sample sizes.

**Methods:**

We assumed that clinical validation study estimates met the ‘desirable’ performance (sensitivity 97%, specificity 99%) in the target product profile (TPP) published by the Medicines and Healthcare products Regulatory Agency (MHRA). To estimate the real-world impact of imprecision imposed by sample size we used Bayesian posterior calculations along with Monte Carlo simulations with 10,000 independent iterations of 5,000 participants. We varied the prevalence between 1 and 15% and the sample size between 30 and 2,000. For each sample size, we estimated the probability that diagnostic accuracy would fail to meet the TPP criteria after implementation.

**Results:**

For a validation study that demonstrates ‘desirable’ sensitivity within a sample of 30 participants who test positive for COVID-19 using the reference standard, the probability that real-world performance will fail to meet the ‘desirable’ criteria is 10.7–13.5%, depending on prevalence. Theoretically, demonstrating the 'desirable' performance in 90 positive participants would reduce that probability to below 5%. A marked reduction in the probability of failure to hit ‘desirable’ specificity occurred between samples of 100 (19.1–21.5%) and 160 (4.3–4.8%) negative participants. There was little further improvement above sample sizes of 160 negative participants.

**Conclusion:**

Based on imprecision alone, small evaluation studies can lead to the acceptance of diagnostic tests which are likely to fail to meet performance targets when deployed. There is diminished return on uncertainty surrounding an accuracy estimate above a total sample size of 250 (90 positive and 160 negative).

**Supplementary Information:**

The online version contains supplementary material available at 10.1186/s41512-021-00116-4.

## Background

Amid the global COVID-19 pandemic caused by the novel coronavirus SARS-CoV-2, there is an urgent need to validate the diagnostic accuracy of new in vitro diagnostics (IVDs) both to increase testing capacity and to increase the availability of rapid tests for different contexts of use. Diagnostic accuracy must be evaluated prior to clinical implementation of each test. As of January 2021, the Foundation for Innovative New Diagnostics (FIND) lists over 1,000 commercially supplied IVDs for COVID-19 [1]. The scale of the clinical validation exercise is therefore vast.

The sample size for a clinical validation study examining diagnostic accuracy is driven by the required precision around the estimates of sensitivity and specificity (i.e. the width of the 95% confidence intervals). However, a plethora of factors beyond precision influence the real-world performance which cannot be understood using evaluation studies alone, due to their artificial nature. An understanding of the translation of results between the evaluation study and real-world setting is essential to avoid implementation of sub-par IVDs.

The desired characteristics of new IVDs for COVID-19 have been stipulated by the Medicine Healthcare products Regulatory Agency (MHRA) in target product profiles (TPPs) [2]. For point of care tests to detect SARS-CoV-2, the MHRA TPP states that the desirable sensitivity of tests is 97% (with a 95% confidence interval (CI) ranging from 93 to 100%), whereas the desirable specificity is 99% (95% CI: 97–100%). However, the MHRA TPP also states that the ‘acceptable’ sensitivity for such tests is 80% (95% CI: 70–100%) and the ‘acceptable’ specificity is 95% (95% CI: 90–100%) [2]. Recent legislation in the United Kingdom requires that IVDs used to facilitate transportation (e.g. air travel) are required to achieve the ‘desirable’ sensitivity and specificity stated in the MHRA TPP when evaluated on 150 ‘positive’ cases (those with COVID-19) and 250 ‘negative’ cases (those without COVID-19) [3].

However, in order to provide ‘derogation’ (the equivalent of emergency use authorization), the Medicines and Healthcare products Regulatory Agency (MHRA) requires companies to provide data from at least 30 positive cases and 30 negative cases. The World Health Organisation (WHO) suggests that new tests for COVID-19 should be evaluated in studies that include at least 250 positive and 1,000 negative cases [4].

These sample sizes differ massively, whilst the optimal choice remains unclear. Clearly, the larger the sample size for a particular evaluation, the greater the certainty surrounding the estimate of accuracy. However, prospective clinical studies with large sample sizes take a long time to reach the recruitment targets, particularly when the prevalence is low or when there is high competition for limited research resource. This increased time is particularly important given the urgent demand for COVID-19 tests during the current pandemic and the large number of tests that still require prospective evaluation.

We aimed to estimate the impact of using evaluations with the different sample sizes, including those specified by the MHRA and WHO, by evaluating the probability that tests will fail to meet target specifications post-implementation, given the clinical validation study met the TPP. We contextualise the results by highlighting the performance under the existing sample size requirements and describing the recruitment rates observed for evaluations of current COVID-19 diagnostic technologies.

## Methods

We evaluated the Bayesian posterior distribution when assuming an uninformative uniform prior, *U* (0, 1), combined with the data observed in the evaluation study. Namely, for an evaluation study with *e*_+_ positive samples and *e*_−_ negative samples, where *n*_+_ positive samples were correctly detected and *n*_−_ negative samples were correctly detected, assuming independent priors, the Bayesian posteriors for sensitivity *α* and specificity *β* are

$$ \alpha \mid {e}_{+},{n}_{+}\sim Beta\left({n}_{+}+1,{e}_{+}-{n}_{+}+1\right), $$

$$ \beta \mid {e}_{-},{n}_{-}\sim Beta\left({n}_{-}+1,{e}_{-}-{n}_{-}+1\right). $$

These posterior distributions were used to obtain theoretical estimates of performance, including the reliability of the true sensitivity/specificity being greater than or equal to the required threshold for passing the evaluation study, given the result observed in the evaluation study. In addition, we conducted a Monte Carlo simulation study in R version 3.6.1 [5] to examine the probability that the real-world performance is below target even though it passed the diagnostic test evaluation. We selected sample sizes for each evaluation ranging from 30 to 2,000 for both positive and negative cases (increasing in increments of 10). We then assumed that the evaluation met or exceeded the MHRA TPP diagnostic accuracy, as these are the only evaluations that should be deployed in the United Kingdom. We considered the scenario where the diagnostic test achieved the minimum accepted performance under the specified TPP since this corresponds to a worst-case scenario. We applied both the MHRA TPP acceptable (sensitivity 80%, 95% CI: 70–100% and specificity 95%, 95% CI: 90–100%) and desirable characteristics (sensitivity 97%, 95% CI: 93–100% and specificity 99%, 95% CI: 97–100%).

Given the assumed values of sensitivity and specificity, we then evaluated the corresponding number of true positives and true negatives that would have been observed in the evaluation sample to obtain these values, rounded up to whole numbers. These informed the parameters of the Beta distributions for sensitivity and specificity from which we estimate the theoretical probability estimates and sample for the simulated population.

The theoretical probability estimates were calculated according to the following:

1. Suppose *p* is the minimum performance threshold for sensitivity (e.g. 97% corresponds to *p* = 0.97) and *e* is the number of positive lab samples in the evaluation study. Then the minimum performance corresponds to correctly identifying ⌈*p* × *e*⌉ of the *e* positive evaluation tests, where ⌈*x*⌉ is the smallest whole number greater than or equal to *x*, often termed the ceiling function.
2. Use this observed performance to determine the posterior distribution of the diagnostic performance measure, e.g. *α*~*Beta*(⌈*p* × *e*⌉ + 1, ⌊(1 − *p*) × *e*⌋ + 1), where ⌊*x*⌋ is the largest whole number less than or equal to *x*, often termed the floor function.
3. Evaluate the mean, 95% confidence interval and reliability from the posterior distribution, i.e.
  $$ \mathrm{Mean}:E\left[\alpha \right]=\frac{\left\lceil p\times \mathrm{e}\right\rceil +1}{e+2} $$

$$ 2.5\mathrm{th}\ \mathrm{percentile}:l\kern0.5em where\ {\int}_0^l\frac{x^{\left\lceil p\times e\right\rceil }{\left(1-x\right)}^{e-\left\lceil p\times e\right\rceil }}{B\left(\left\lceil p\times e\right\rceil +1,e-\left\lceil p\times e\right\rceil +1\right)} dx=0.025 $$

$$ 97.5\mathrm{th}\ \mathrm{percentile}:u\kern0.5em where\ {\int}_0^u\frac{x^{\left\lceil p\times e\right\rceil }{\left(1-x\right)}^{e-\left\lceil p\times e\right\rceil }}{B\left(\left\lceil p\times e\right\rceil +1,e-\left\lceil p\times e\right\rceil +1\right)} dx=0.975 $$

$$ \mathrm{Reliability}:P\left(\alpha \ge p|\kern0.5em \left\lceil p\times e\right\rceil\ correctly\ detected\ out\ of\ e\  positive\  lab\  samples\right)={\int}_p^1\frac{x^{\left\lceil p\times e\right\rceil }{\left(1-x\right)}^{e-\left\lceil p\times e\right\rceil }}{B\left(\left\lceil p\times e\right\rceil +1,e-\left\lceil p\times e\right\rceil +1\right)} dx $$

where $B\left(a,b\right)=\frac{\Gamma (a)\Gamma (b)}{\Gamma \left(a+b\right)}$, *Γ* is the gamma function and α is the true sensitivity. Similar equations are derived for the specificity where *e* represents the number of negative lab samples and *p* is the minimum performance for the specificity aspect of the TPP.

For the Monte Carlo simulations, we simulated the disease status for each individual in the population according to a specific value of prevalence using a binomial distribution. These were dichotomised into true positives/false negatives and true negatives/false positives according to the binomial distributions using the simulated values of the sensitivity and specificity, respectively. From this, we calculated the simulated real-world sensitivity and specificity estimates of the diagnostic test. Details for the simulation procedure in which a diagnostic test was assumed to have met the desirable TPP are given in Algorithm 1 and the R code is available at https://github.com/csammutpowell/EvaluationSampleSize.

This simulation was run for real-world scenarios of 5,000 patients 10,000 times per sample size per prevalence scenario. This simulation size was calculated to be sufficient for a type I error of < 5% for detecting a difference outside the desirable TPP confidence interval, assuming a 1% prevalence. The described variations of the different TPPs (acceptable and desirable) resulted in two simulation settings (Table 1).

**Table 1:** Description of the simulation setting assumptions made based on the Target Product Profiles (TPPs), where *D*_min_ corresponds to the scenario where the minimum number of cases were correctly detected to satisfy the desirable TPP and *A*_min_ corresponds to the scenario where the minimum number of cases were correctly detected to satisfy the acceptable TPP

Setting	Setting 1 (*D*_min_)	Setting 2 (*A*_min_)
*N* (simulation population size)	5,000	5,000
Iterations	10,000	10,000
Evaluation performance assumption	Equal to desirable characteristics (97% sensitivity and 99% specificity)	Equal to acceptable characteristics (80% sensitivity and 95% specificity)
Failure criterion	less than the lower bound of 95% confidence interval (93% sensitivity and 97% specificity)	less than the lower bound of 95% confidence interval (70% sensitivity and 90% specificity)

We modelled sensitivity and specificity independently and ran our simulation across multiple prevalence scenarios (1%, 5%, 10%, 15%) representative of those observed nationally and within hospital presentations. We highlight the results corresponding to evaluation sample sizes suggested in current guidelines: MHRA derogation (30 positive/30 negative), MHRA TPP (150 positive/250 negative) and WHO (250 positive/1,000 negative).

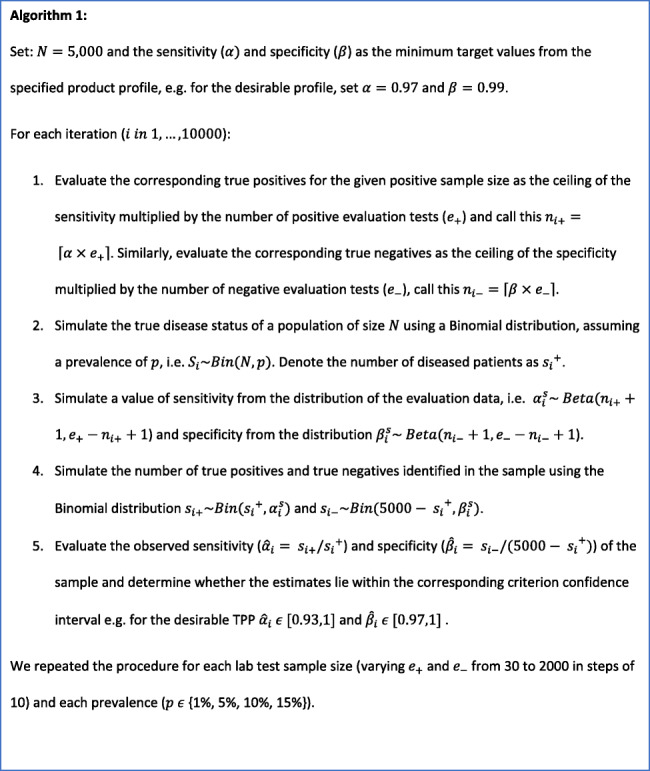



To evaluate the real-world impact of introducing new tests following evaluations with each of those sample sizes, we report the 95% confidence intervals for the estimates of sensitivity and specificity and the reliability i.e. the posterior probability that the true sensitivity and specificity are greater than or equal to the minimum accepted performance for the specified TPP. We compared this with the lower bound of the 95% Wilson interval. We also report the change in the lower bound of the estimated 95% confidence intervals as this parallels the poorest expected performance in practice. Further, we report the proportion of estimates that failed to meet the TPP with an observed performance outside the corresponding TPP confidence interval, i.e. for the desirable TPP, a simulation with a sensitivity below 93% would fail to meet the TPP. These proportions represent the probability that an approved test would fail to meet the TPP in practice, herein termed 'failure'.

## Results

The theoretical performances indicate that evaluations with fewer than 100 cases have much wider confidence intervals and a high probability that the true performance is below the lower bound of the desirable TPP confidence interval. For specificity, the probability of failure when using only 30 cases is 38.9%, even though no errors can be observed in order to pass the evaluation. This is considerably reduced to 1.9% by increasing the number of negative cases to 250. A similar pattern is observed for sensitivity, with a 10.9% probability of failure in 30 positive cases compared to 1.7% in 150 positive cases.

**Fig. 1:**
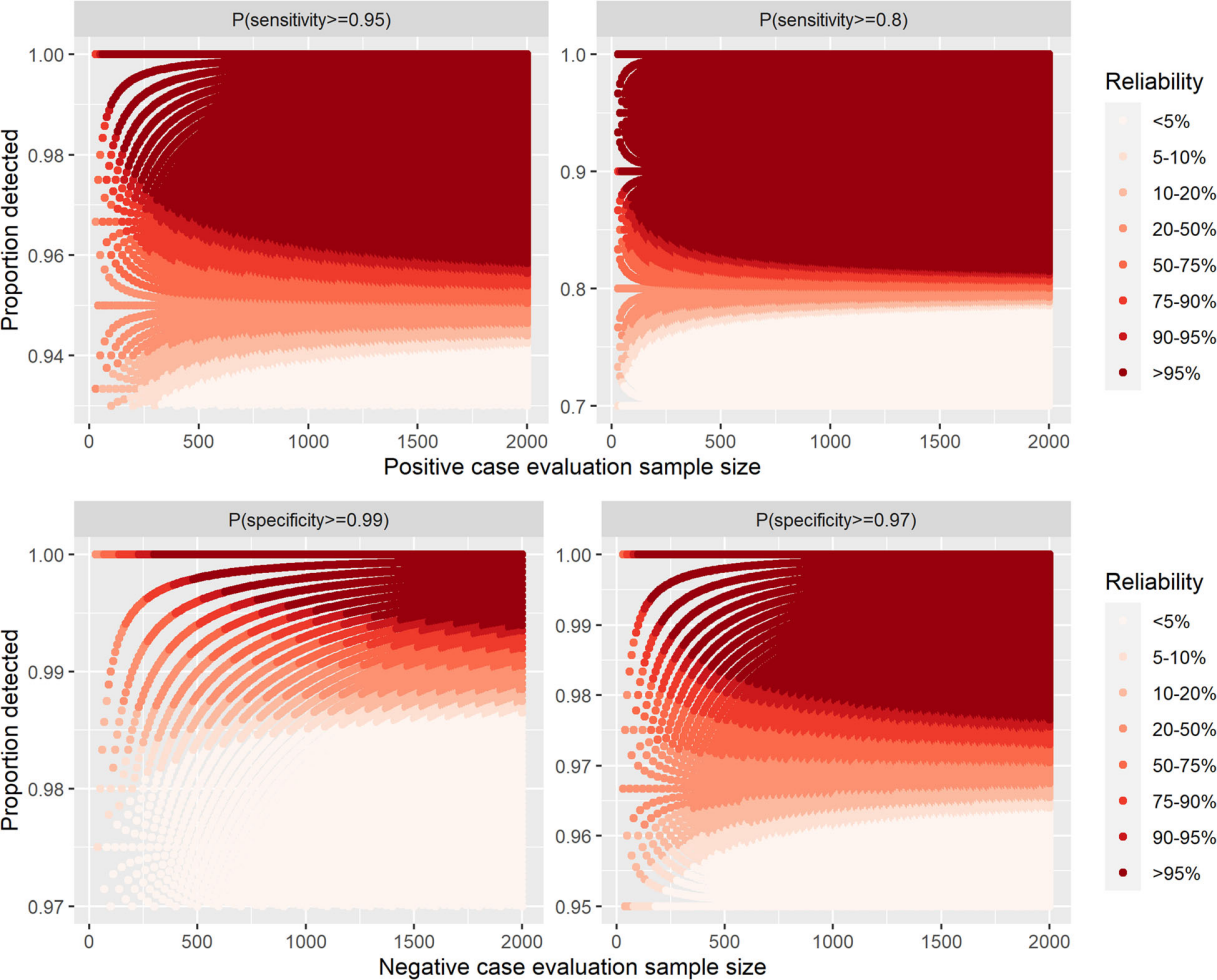
Reliability for each of the Target Product Profiles (TPPs), where the reliability is the probability that the true value of the performance measure is greater than or equal to the TPP, given the result of the proportion of samples detected within a specified evaluation study sample size

Further, our results demonstrate that the minimum number of negative evaluation samples needed to achieve a probability of at least 0.95 that the true specificity is equal to or exceeds 99% and 97% are 300 and 100, respectively (Fig. 1). Similarly, the minimum number of positive evaluation samples to achieve at least 0.95 probability that the true sensitivity is at least 95% and 80% are 60 and 30, respectively (Fig. 1). Across these circumstances, only in the latter case is there room for misclassification within the specified evaluation sample size; for the evaluations relating to specifity, 100% detection is required for the reliability to be above 0.95 under these sample sizes. This was reiterated from the plots comparing reliability with the lower bound of the 95% Wilson interval estimated from the observed data (Supplementary Fig. S1).

**Fig. 2:**
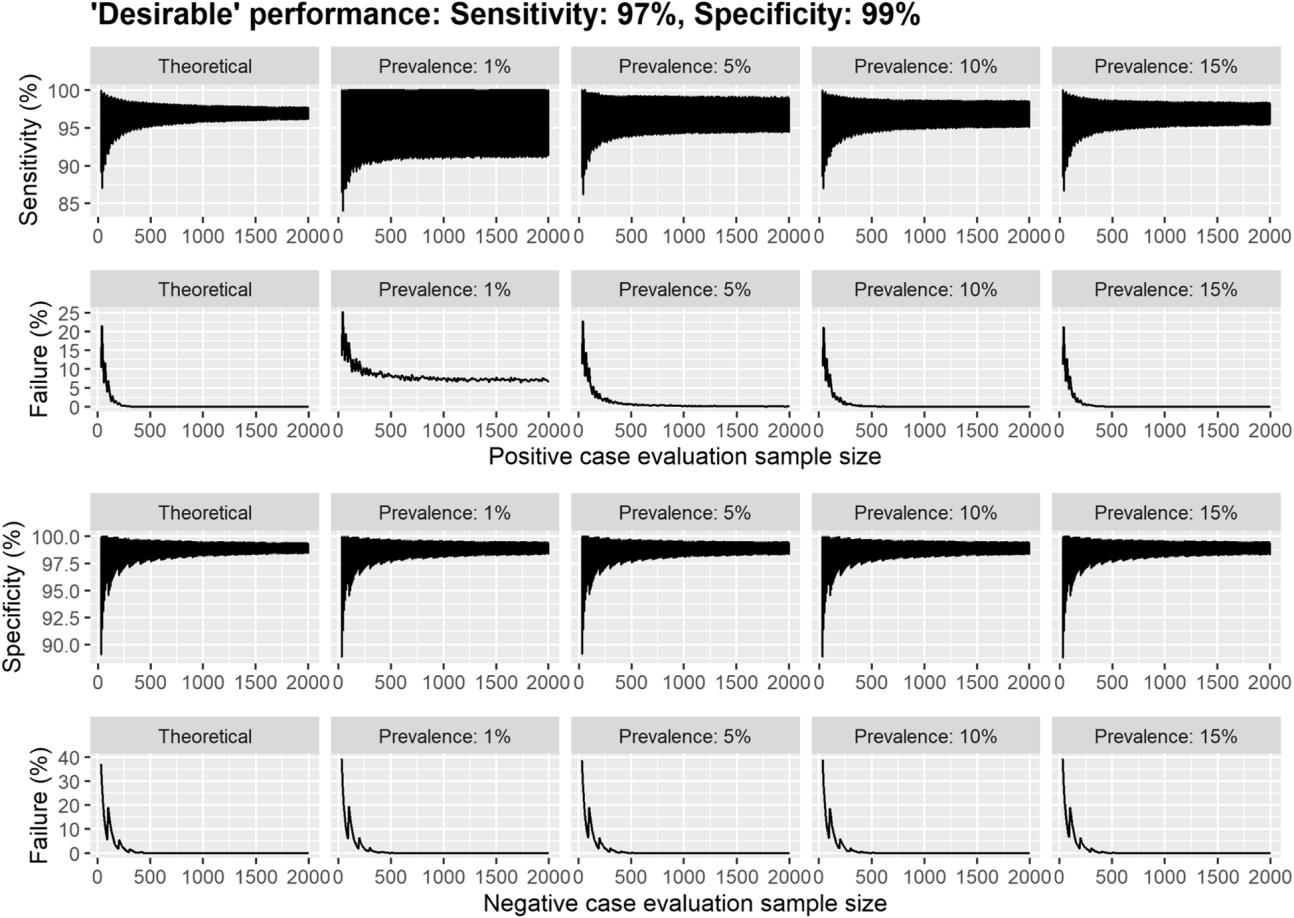
*D*_min_ scenario estimates of diagnostic performance from a series of Monte Carlo simulations per evaluation sample size. Each simulation consisted of 10,000 iterations each consisting of 5,000 individuals. Here the diagnostic test was assumed to be greater than or equal to the performance for the desirable MHRA TPP with 97% sensitivity and 99% specificity in the evaluation sample. The 95% confidence intervals are displayed for sensitivity and specificity per initial evaluation sample size across different prevalence scenarios. The simulation was considered to have met the TPP confidence interval criterion if the diagnostic characteristic was above the lower bound of the 95% CI specified in the TPP (93% sensitivity and 97% specificity)

Due to the fixed simulation sample size, the simulated ‘real-world’ 95% confidence intervals for sensitivity were affected by the prevalence of the disease, with the confidence interval shrinking as the prevalence increased (Fig. 2 and Table 2). In particular, in the scenario assuming a prevalence of 1%, the final sample size corresponds to a mean of 50 positive cases in the population, therefore any inaccuracy corresponds to a larger proportion than in other prevalence scenarios. This influences both the confidence intervals and probability of failure. For example, when evaluating the performance of a study with 2,000 ‘positive’ participants (with COVID-19) in Setting 1 (*D*_min_), the lower bound of the 95% confidence interval for sensitivity was approximately 91% for a prevalence of 1%, whereas it was over 95% for a prevalence of 15% (Table 2). The corresponding results for the acceptable TPP (*A*_min_) are detailed in Supplementary Table S1.

**Table 2:** *D*_min_ scenario estimated mean and 95% confidence intervals of the sensitivity and specificity and the proportion that failed to meet the lower bound of the desirable TPP criteria confidence interval (sensitivity 93%; specificity 97%) assuming the test achieved minimum performance for the desirable TPP (*D*_min_: 97% sensitivity and 99% specificity) in the evaluation sample, for a simulated population size of 5,000

Evaluation sample size	Observed TP/TN in evaluation	Theoretical				Simulation															
						Prevalence: 1%				Prevalence: 5%				Prevalence: 10%				Prevalence: 15%			
		Mean	95% CI		Failure (%)	Mean	95% CI		Failure (%)	Mean	95% CI		Failure (%)	Mean	95% CI		Failure (%)	Mean	95% CI		Failure (%)
Sensitivity																					
30	30	96.9%	88.8%	99.9%	10.5%	96.9%	86.5%	100.0%	13.5%	96.9%	88.5%	100.0%	11.3%	96.9%	88.5%	100.0%	10.7%	96.9%	88.5%	100.0%	11.1%
50	49	96.2%	89.6%	99.5%	11.9%	96.2%	86.8%	100.0%	17.7%	96.1%	88.8%	100.0%	14.0%	96.1%	89.3%	99.6%	13.2%	96.2%	89.3%	99.6%	12.4%
100	97	96.1%	91.6%	98.9%	7.1%	96.1%	87.8%	100.0%	17.2%	96.0%	90.6%	99.5%	10.3%	96.1%	91.2%	99.2%	8.6%	96.1%	91.3%	99.1%	8.1%
150	146	96.7%	93.4%	98.9%	1.7%	96.8%	89.8%	100.0%	10.7%	96.7%	92.5%	99.6%	3.7%	96.7%	92.8%	99.2%	2.9%	96.7%	93.1%	99.1%	2.3%
200	194	96.5%	93.6%	98.6%	1.1%	96.5%	89.6%	100.0%	12.1%	96.5%	92.7%	99.2%	3.5%	96.5%	93.0%	99.0%	2.6%	96.5%	93.3%	98.8%	1.8%
250	243	96.8%	94.3%	98.6%	0.3%	96.8%	90.4%	100.0%	9.6%	96.8%	93.1%	99.2%	2.3%	96.8%	93.7%	99.0%	0.9%	96.8%	93.9%	98.9%	0.6%
500	485	96.8%	95.1%	98.2%	0.0%	96.8%	90.7%	100.0%	8.4%	96.8%	93.8%	99.2%	0.8%	96.8%	94.3%	98.8%	0.2%	96.8%	94.6%	98.6%	0.1%
1000	970	96.9%	95.7%	97.9%	0.0%	96.9%	91.1%	100.0%	7.5%	96.9%	94.3%	99.1%	0.3%	96.9%	94.9%	98.6%	0.0%	96.9%	95.1%	98.4%	0.0%
2000	1940	97.0%	96.2%	97.7%	0.0%	97.0%	91.4%	100.0%	6.9%	97.0%	94.4%	98.9%	0.2%	96.9%	95.1%	98.5%	0.0%	97.0%	95.4%	98.3%	0.0%
Specificity																					
30	30	96.9%	88.8%	99.9%	38.9%	96.9%	88.8%	99.9%	39.5%	96.9%	89.1%	99.9%	38.7%	96.9%	88.9%	99.9%	39.1%	96.9%	88.8%	99.9%	39.5%
50	50	98.1%	93.0%	100.0%	21.2%	98.1%	93.1%	100.0%	20.1%	98.1%	93.1%	100.0%	21.7%	98.1%	92.9%	100.0%	21.5%	98.1%	93.0%	100.0%	20.7%
100	99	98.0%	94.6%	99.8%	19.0%	98.0%	94.5%	99.8%	19.4%	98.0%	94.7%	99.8%	19.1%	98.1%	94.5%	99.8%	18.5%	98.0%	94.5%	99.8%	18.9%
150	149	98.7%	96.4%	99.8%	5.7%	98.7%	96.4%	99.9%	5.6%	98.7%	96.4%	99.9%	5.8%	98.7%	96.4%	99.9%	6.1%	98.7%	96.3%	99.9%	5.7%
200	198	98.5%	96.5%	99.7%	5.8%	98.5%	96.4%	99.7%	6.3%	98.5%	96.4%	99.7%	6.4%	98.5%	96.4%	99.7%	6.0%	98.5%	96.4%	99.7%	6.3%
250	248	98.8%	97.2%	99.8%	1.9%	98.8%	97.1%	99.8%	2.1%	98.8%	97.1%	99.8%	2.2%	98.8%	97.1%	99.8%	2.0%	98.8%	97.1%	99.8%	2.3%
500	495	98.8%	97.7%	99.6%	0.2%	98.8%	97.6%	99.6%	0.3%	98.8%	97.6%	99.6%	0.3%	98.8%	97.7%	99.6%	0.3%	98.8%	97.6%	99.6%	0.4%
1000	990	98.9%	98.2%	99.5%	0.0%	98.9%	98.1%	99.5%	0.0%	98.9%	98.1%	99.5%	0.0%	98.9%	98.1%	99.5%	0.0%	98.9%	98.1%	99.5%	0.0%
2000	1980	99.0%	98.5%	99.3%	0.0%	98.9%	98.3%	99.4%	0.0%	98.9%	98.4%	99.4%	0.0%	98.9%	98.4%	99.4%	0.0%	99.0%	98.3%	99.4%	0.0%

TP = true positives (in sensitivity), TN = true negatives (in specificity)

**Fig. 3:**
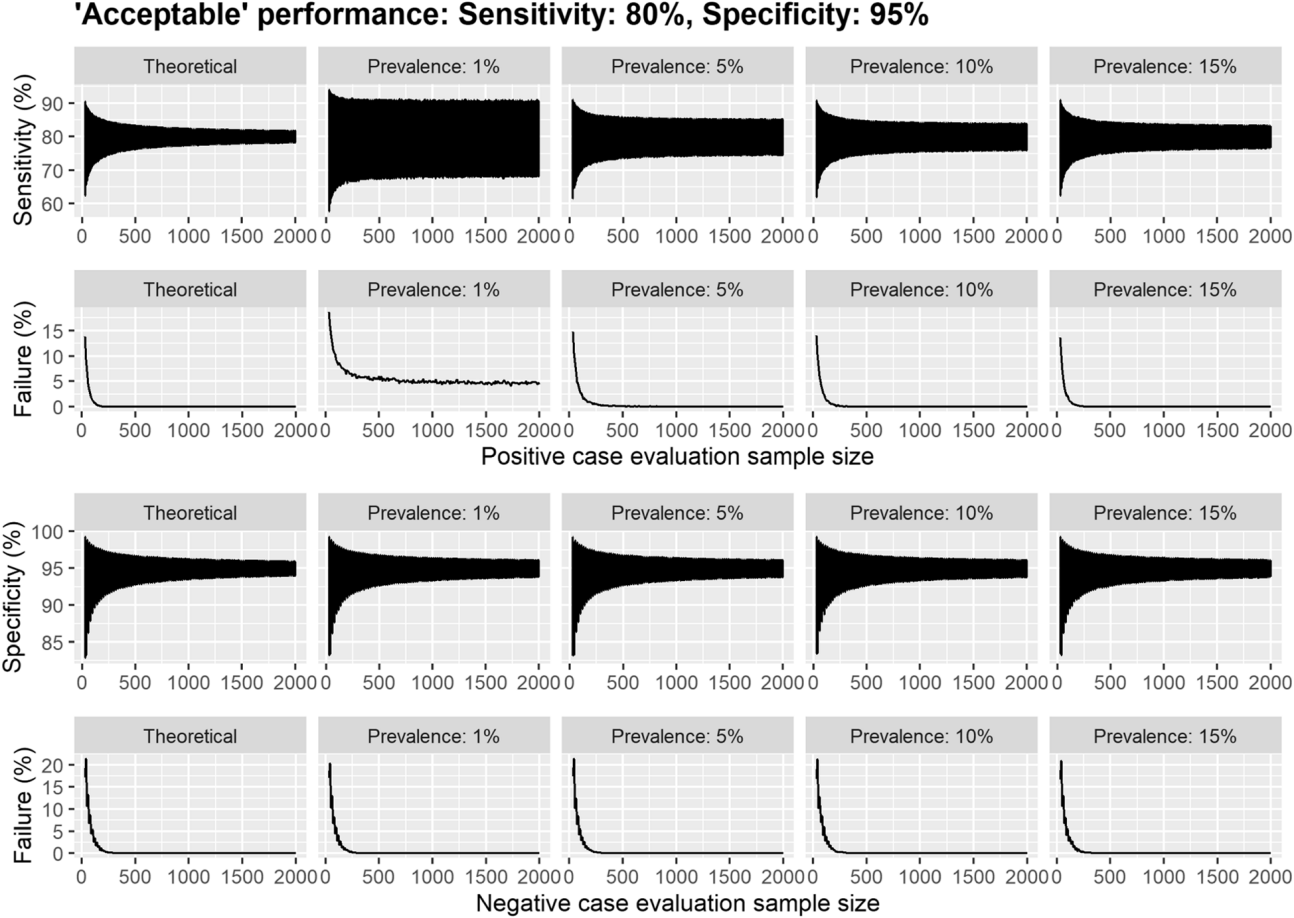
*A*_min_ scenario estimates of diagnostic performance from a series of Monte Carlo simulations per evaluation sample size. Each simulation consisted of 10,000 iterations each consisting of 5,000 individuals. Here, the diagnostic test was assumed to be greater than or equal to the minimum performance for the acceptable MHRA TPP with 80% sensitivity and 95% specificity in the evaluation sample. The 95% confidence intervals are displayed for sensitivity and specificity per initial evaluation sample size across different prevalence scenarios. The simulation was considered to have met the TPP confidence interval criterion if the diagnostic characteristic was above the lower bound of the 95% CI specified in the TPP (70% sensitivity and 90% specificity)

Prevalence has a marked impact on the probability of ‘failure’, i.e. the proportion of simulations in which a test had shown at least ‘desirable’ sensitivity and/or specificity in the study, but failed to achieve ‘desirable’ performance once implemented, when the simulation sample size is fixed (Figs. 2 and 3 & Supplementary Figs. S2 and S3). For example, at a prevalence of 1%, the probability of ‘failure’ improved only marginally (from 13.5% to 10.7%) after increasing the sample size from 30 to 150 ‘positive’ participants. However, when the prevalence was 10%, there was a marked improvement (from 10.7% to 2.9%).

In the scenario with an assumed sensitivity of 97% and specificity of 99% (MHRA desirable TPP), the theoretical confidence interval with the smallest lower bound and highest probability of failure occurs at the sample size of 40. This is because to achieve a 97% sensitivity with 30 samples, there cannot be any incorrectly classified results. However, when this is increased to 40, a sensitivity of 97% requires to 39/40 to be correctly specified. Consequently, this corresponds to a smaller observed sensitivity in the evaluation tests, which is reflected in the confidence interval and inflated probability of failure.

Considering sample size for ‘negative’ cases (patients without COVID-19), there was a substantial reduction in the probability of ‘failure’ in specificity between sample sizes of 30 ‘negatives’ (theoretical: 38.9%, simulated: 39.1–39.5%) and 160 ‘negatives’ (theoretical: 4.4%, simulated: 4.3–4.8%). Increasing sample size had a similar effect on the 95% confidence intervals.

A consistent pattern was observed when increasing the evaluation sample size; an elbow was observed in the plots when the evaluation sample size was approximately 250. The elbow occurred in both the 95% confidence intervals and in the proportion that failed to meet the assumed TPP. Beyond this point, there was little improvement across all performance measures. This was apparent across all simulation settings and fewer than 1% of the estimates for sensitivity and specificity failed to meet the assumed TPP, except for sensitivity when the prevalence is assumed to be 1%. Supplementary Figs. S2 and S3 demonstrate the changes observed for evaluation sample sizes between 30 and 100 across all scenarios.

If we consider the competing guidelines in practice, we observed that there was a considerable difference in the expected performance in the population, particularly in the lower bounds when the prevalence was low (Table 3). Across all assumed values for the prevalence, the lower bound increased by 9 percentage points between the smallest (30/30) and medium (150/250) sample size guidelines under setting 1 (*D*_min_). There was also a considerable reduction in the probability of failure to meet the TPP for specificity, changing from 39% to 2%. When evaluating the performance under the minimum acceptable diagnostic characteristics as per the MHRA TPP (target sensitivity 80% and specificity 95%), we observed that the sensitivity in the simulated population could be as low as 57.7% for a prevalence of 1% (Supplementary Table S2) under the smallest sample size guidelines. The change in performance with evaluation sample size was similar to those observed for the ‘desirable’ rates; however, the confidence intervals often contained what may be considered unacceptable performances (Fig. 2 & Supplementary Fig. S2). Our results suggest that to achieve a probability of failure below 5%, a split of (90/160) may be sufficient (Supplementary Table S3).

**Table 3:** *D*_min_ scenario estimated mean and 95% confidence intervals of the sensitivity and specificity of a diagnostic test, given the test achieved 97% sensitivity and 99% specificity in the evaluation sample (*D*_min_), for a simulated population size of 5,000, where the evaluation sample sizes correspond to current guidelines

Evaluation sample size	Sensitivity				Specificity			
Positive (*e*_+_) /negative (*e*_−_)	Mean	95% CI		Failure (%)	Mean	95% CI		Failure (%)
Theoretical								
30/30	96.9%	88.8%	99.9%	10.5%	96.9%	88.8%	99.9%	38.9%
150/250	96.7%	93.4%	98.9%	1.7%	98.8%	97.2%	99.8%	1.9%
250/1000	96.8%	94.3%	98.6%	0.3%	99.0%	98.5%	99.3%	0.0%
Prevalence: 1%								
30/30	96.9%	86.5%	100.0%	13.5%	96.9%	88.8%	99.9%	39.5%
150/250	96.8%	89.8%	100.0%	10.7%	98.8%	97.1%	99.8%	2.1%
250/1000	96.8%	90.4%	100.0%	9.6%	98.9%	98.1%	99.5%	0.0%
Prevalence: 5%								
30/30	96.9%	88.5%	100.0%	11.3%	96.9%	89.1%	99.9%	38.7%
150/250	96.7%	92.5%	99.6%	3.7%	98.8%	97.1%	99.8%	2.2%
250/1000	96.8%	93.1%	99.2%	2.3%	98.9%	98.1%	99.5%	0.0%
Prevalence: 10%								
30/30	96.9%	88.5%	100.0%	10.7%	96.9%	88.9%	99.9%	39.1%
150/250	96.7%	92.8%	99.2%	2.9%	98.8%	97.1%	99.8%	2.0%
250/1000	96.8%	93.7%	99.0%	0.9%	98.9%	98.1%	99.5%	0.0%
Prevalence: 15%								
30/30	96.9%	88.5%	100.0%	11.1%	96.9%	88.8%	99.9%	39.5%
150/250	96.7%	93.1%	99.1%	2.3%	98.8%	97.1%	99.8%	2.3%
250/1000	96.8%	93.9%	98.9%	0.6%	98.9%	98.1%	99.5%	0.0%

## Discussion

This study provides an understanding of the effect of evaluation sample size on real-world performance of a diagnostic study. Our key findings were that 1) there was little benefit to having a sample size above 250 and the probability of failure to meet desirable guidelines can be reduced to below 5% using 90 positives and 160 negatives; 2) when a diagnostic test performs with 100% accuracy in 30 positive cases, there is still > 10% chance that the sensitivity will be below 93% and the > 38% chance the specificity will be below 97% in a real-world setting.

There is a clear difference in the sensitivity when measured in the real-world setting compared to the evaluation results. The simulations imply that when prevalence of COVID-19 in the population is high, the probability that an IVD will fail to achieve desirable/acceptable sensitivity after implementation is smaller when comparing across a fixed sample size (Fig. 3); however, this is an artefact of the fixed sample size and disappears when larger evaluation and ‘real-life’ samples are used (see Supplementary Table S5). Hence, if a test is re-evaluated in the target setting, the sample size needs to be sufficient to make a fair comparison with the theoretical performance.

In any research, having a sufficient sample size is crucial to obtain valid results [6–8]. However, it can be difficult to obtain large samples due to several reasons. In the COVID-19 pandemic, speed is a crucial component to a successful response [9, 10] but this comes at a cost. This has been observed within the prediction modelling community, where a lot of research rapidly published has been deemed to be at a high risk of bias, including diagnostic prediction models [11]. Key features which were identified amongst these studies were small sample sizes and the use of samples that were not representative of the target population. Evaluations of diagnostic tests are no exception to these.

Under the current emergency guidelines from the MHRA, companies are required to evaluate diagnostic tests in 30 positive and 30 negative cases. To achieve the desirable target product profile with a sensitivity of 97% and specificity of 99%, this requires all 60 cases to be correctly identified. However, we have shown that even when this is achieved, the observed sensitivity of the test in a real-world scenario could be as low as 86.5%, with a > 10% chance of being below 93%. Similarly, the specificity could be as low as 88.8%, with a > 38% chance of being below 97%. This is assuming the evaluation samples are representative, hence when this is not the case, we could expect a poorer performance. In practice, obtaining this level of performance in 30 samples could be rare, and consequently, the adequate performance measures offer a more lenient benchmark. Should a test perform with 80% sensitivity and 93% specificity in 30 positive and negative samples, respectively, their real-world sensitivity and specificity could be as low as 57.7% and 83.2%, respectively. These drops in performance were universal across the assumed values of prevalence and careful consideration is required to re-assess if this is truly acceptable.

There is a diminishing return on increasing the evaluation sample size to improve the certainty of an assay’s in-practice diagnostic characteristics, particularly when time is of the essence. We observed an exponential decrease in uncertainty (narrowing 95% confidence intervals), across prevalence for both sensitivity and specificity up to 250 cases (for sensitivity, 250 positive cases: 95% CI 94.3%-98.6% vs 2,000 positive cases: 95% CI 96.2%-97.7%). Consequently, 250 may be the optimal sample size when the cases are representative of those in the target population and case recruitment is rapid enough. This optimal sample size is corroborated by theoretical sample size calculations for diagnostic studies presented in Bujang & Adnan 2016 [12]. However, smaller sample sizes of 90 positives and 160 negatives demonstrated a probability of failure of less than 5% to meet the TPP, hence may be sufficient when larger samples are unreasonable.

Our simulation study approach provides an accessible understanding of how the evaluation sample size influences the expected real-world performance and is generalisable beyond the COVID-19 application to all diagnostic evaluation studies. Although a formal sample size calculation could be conducted to determine the minimum sample size [12, 13], it requires defining the statistical power, type I error and effect size. These are not always easy to decide upon, nor provide the context around confidence intervals of performance that would be expected. Our approach to calculate sample size based on precision is a recognised alternative [14] that allows for an understanding of the performance in sub-optimal samples which is likely to occur in these kinds of evaluations due to limited resource and competing recruitment. Further, we were able to evaluate how a test which passed the criteria (an observed value greater than or equal to the minimum accepted value) would be expected to perform. Such scenarios are not well defined in the theoretical sample size calculation realm. By taking our simulation approach, we were also able to identify interesting features within small samples. For example, we observed an anomaly where the confidence intervals of sensitivity increased in size from a sample size of 30 to 40 positive cases (*e*_+_). This is because when *e*_+_ = 30 it is not possible to achieve the desirable sensitivity with any false negatives however when *e*_+_ = 40 you can observe one. This highlights one difficulty of arbitrary small sample sizes, where it can give a false impression of poor diagnostic accuracy.

The Facilitating AcceLerated Clinical Validation Of Novel Diagnostics for COVID-19 (FALCON-C19) study is a diagnostic platform, designed to facilitate clinical validation of multiple IVDs for COVID-19. The study has been prioritised for delivery during the pandemic as part of the National Institute for Health Research Urgent Public Health portfolio and has opened to recruitment at 67 hospital sites and 16 community testing centres across the United Kingdom. Recruitment data from FALCON C-19 can be used to contextualise the simulation sample sizes (see ‘Supplementary methods’ and ‘Supplementary results’ in the Supplementary materials). Evaluations run within the hospital settings suggest that it could take between 98 and 408 days to reach a sample size of 150 positive and 250 negative cases (Supplementary Table S4). However, a better understanding of the recruitment strategy is needed to formally assess an expected time. For example, it may be possible to reduce this time with additional measures and resource to obtain samples. Experience with FALCON-C19 suggests that recruitment rates are likely to be faster at community testing centres (Supplementary Fig. S3).

The need to evaluate multiple IVDs during the pandemic also presents a unique challenge. Recruitment to several evaluations in parallel will slow overall recruitment rates for each one, whereas recruitment in series would limit the number of IVDs that can be evaluated in a given timescale. A practical approach could be to perform interim analyses to determine whether the tests are performing sufficiently well to justify continuing their evaluation. This parallels with the well-established concept of adaptive designs. It could avoid wasting resources when diagnostic tests are considerably underperforming, allowing the redirection of resource to evaluations of more promising diagnostic tests. For example, there are regions of performance which can be identified across various sample sizes to determine if the probability of failure to achieve a given performance threshold is below 5% (Supplementary Figs. S5 and S6) and it may be reasonable to conclude sufficient performance early. Similarly, it may be possible to conclude when continuing an evaluation is futile, given a target performance in the maximum sample size.

## Limitations

The nature of simulation study provides us with a known disease status under an assumed prevalence. However, this information is unlikely to be available in practice. Consequently, a joint model between prevalence, sensitivity and specificity may be used to estimate sensitivity and specificity in practice which could lead to different results.

Within the simulations, we made several assumptions. Firstly, we assumed a fixed sample size of 5,000; however, when the prevalence is 1%, this corresponds to a small ‘positive’ sample, resulting in a convergence to a larger failure rate than observed in other scenarios. This prevalence corresponds to a national scale, rather than those that would be observed within the hospital setting; hence, it might be more appropriate to consider a larger simulation sample. In larger samples, we can observe similar patterns to those observed for a larger prevalence in this simulation (Supplementary Table S5). Secondly, we considered the minimum performance required to pass the TPP criteria. Therefore, our simulation results are potentially pessimistic in the context of all tests which pass the TPP.

For simplicity, we have not differentiated between evaluation studies which are analytical (often using stored or spiked samples) and clinical studies where the samples are prospectively collected, nor their statistical study design. The performance estimates are likely to be more robust if generated from the latter type which will affect how close these accuracy values are to the in-practice performance. Even with an extremely large sample size, an evaluation which does not reflect the correct target population or clinical context of use could generate estimates of accuracy which differ widely from the real-world in practice accuracy, implying that the results within our study could be considered optimistic. This may be evident when applying a single IVD across multiple case-mix scenarios. Furthermore, we have assumed that negative and positive samples can be collected in isolation. Evaluations are sometimes conducted in known positive and known negative cases, where our method would be directly applicable. However, best practice is for the evaluation to be conducted in the population in which it is to be used [15]. In this case, the target population would be suspected cases, where it is not known if they are positive or negative. The application of our simulation’s findings to this scenario would likely result in the over recruitment of negative participants whilst positive cases are sought (with a prevalence < 0.5), therefore resulting in the positive case sample size being the pragmatic target.

In estimated time to recruitment, we have inferred that the recruitment rate was linear for simplicity. We did however observe a ‘warm up’ period where sites became familiar with the technology and the recruitment rate increased in the first 2 weeks. The complexities of analysing recruitment data are vast and are beyond this scope of work. However, it will be affected by different site sizes, local prevalence rates, local priorities and local resources.

## Conclusions

Increasing the validation sample size results in a greater confidence in the real-world diagnostic characteristics of the assay, under important assumptions. However, there are diminishing returns of increasing beyond a sample size of 250 positive and 250 negative participants. It may be sufficient to observe a desirable performance in 90 positives and 160 negatives to expect to meet the desirable TPP in practice. A staged recruitment with interim analyses could prevent wasting resource.

Hospital recruitment is eclipsed by the fast pace of recruitment at test and trace centres; however, an assay should, ideally, be tested in the setting in which it will be deployed. Hence there must be caution if a diagnostic technology for the hospital is validated in the community setting. When resources are limited, a pragmatic approach is to consider interim analysis to determine whether to continue or cease allocating resources to the validation according to the small sample performance.

## Supplementary Information

**Additional file 1.** Supplementary Material [16].

## Data Availability

The R code for the simulation is available on request.
