# Supplementary material for: Examining the effect of evaluation sample size on the sensitivity and specificity of COVID-19 diagnostic tests in practice: a simulation study
Source: Diagn Progn Res. 2022 Apr 25;6:12. doi: 10.1186/s41512-021-00116-4 (PMC9035779; doi:10.1186/s41512-021-00116-4)
Supplement: Supplementary file 1 — Additional file 1. Supplementary Material [16]. [file 41512_2021_116_MOESM1_ESM.pdf]

Examining the effect of evaluation sample size on the sensitivity and specificity of COVID-19 diagnostic tests in practice: A simulation study

Supplementary Material

Supplementary Methods

We provide an estimate for the time taken to complete evaluations within the Facilitating AcceLerated Clinical Validation Of Novel Diagnostics for COVID-19 (FALCON-C19) study. The evaluations consisted of: a community test and trace evaluation (the moonshot evaluation), a hospital point of care (POC) evaluation, and two hospital sample collection evaluations (A and B)[1]. The moonshot evaluation focused on the recall of positive cases identified from NHS community test and trace centres (symptomatic positive community cases)[1]. It solely examined lateral flow devices but required significant National Institute for Health Research (NIHR) Clinical Research Network (CRN) research nurse support to conduct telephone consent and then onsite nurses to run the tests. The POC evaluation took place in hospitals and incorporated positive and negative cases, the device was tested in situ at the patient's bedside. It had a run time of 12 mins and a preparation time of approximately 5 minutes. The hospital evaluations were supported by local secondary care research nurses who were locally allocated to studies based on national prioritisation. Both hospital evaluations involved the collection of specimens from the patient that were then sent to external laboratories for evaluation on novel technologies offsite. It was assumed that the hypothetical evaluation took part in the same physical, prevalence and resource setting. We considered the recruitment to be linear and calculated a rate from the sum of recruits and the duration of recruitment for both positive and negative cases.

Supplementary Results

The FALCON-C19 study moonshot evaluation lasted 6 weeks, across 15 test and trace sites and recruited 880 COVID-19 positive participants. The POC evaluation lasted 11 weeks across 7 sites and recruited 403 negative participants and 118 positive participants. Both hospital sample collection evaluations involved the collection of specimens from the patient that were then sent to external laboratories for evaluation on novel technologies offsite. For the evaluations that involved specimen collection, evaluation A ran for 17 weeks across six sites and recruited 94 positive cases and 147 negative cases and evaluation B ran for 17 weeks across seven sites and recruited 65 positive participants and 73 negative participants. The recruitment rates for positive and negative cases across the different evaluations are visualised in Figure S4. Moonshot demonstrated a much faster rate of positive case recruitment, than any other evaluation. Consequently, it was found to outperform the other evaluations in time to completion whilst the hospital sample collection evaluations require significant amounts of time more than 5 years to complete the largest samples size (Table S4). Interestingly the other evaluations only saw a slight increase in recruitment rate with increasing national prevalence of COVID-19.

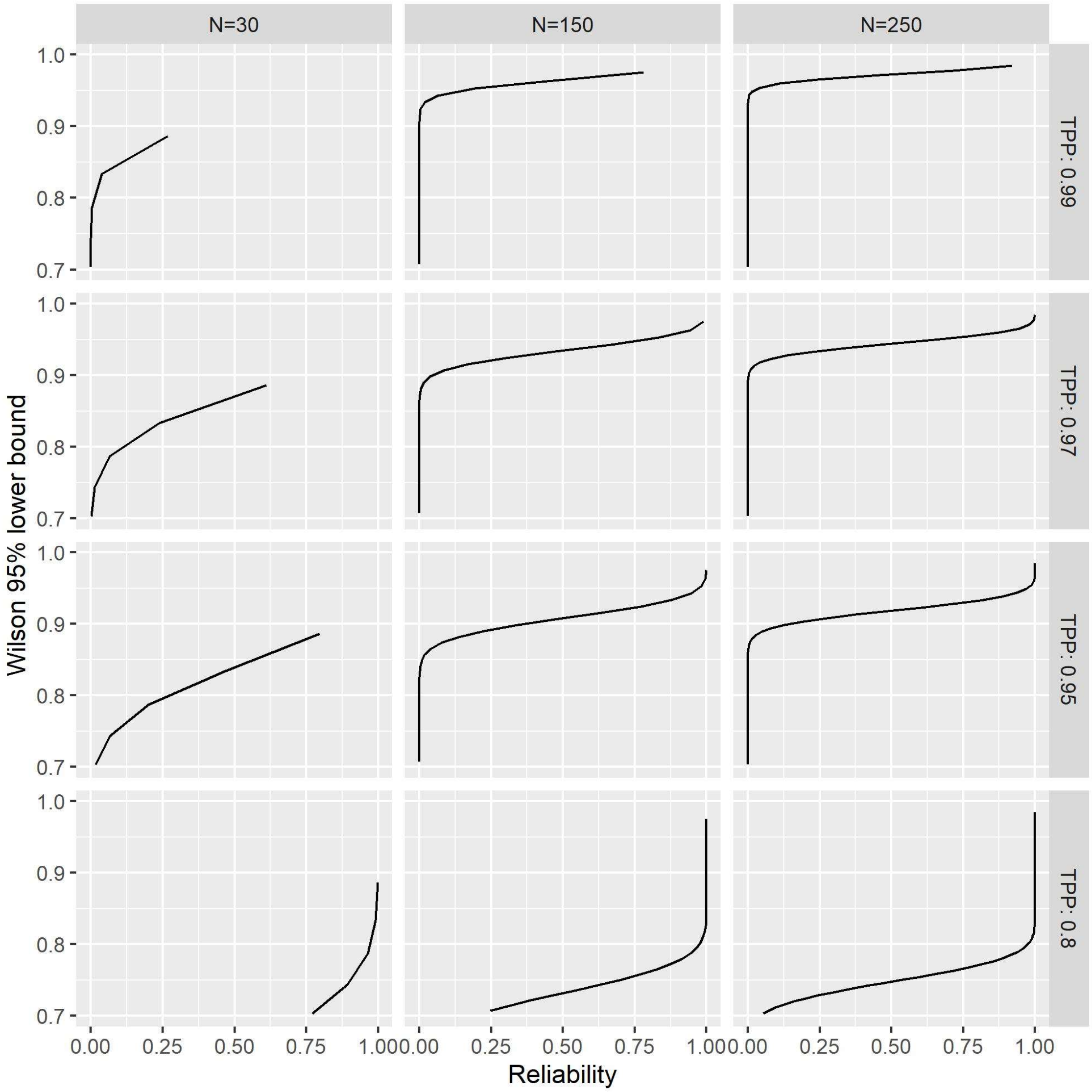

Supplementary Figure S1: Reliability and Wilson 95% lower bound across observed outcomes in the evaluation study, for ech of the target product profiles for the evalutation sizes of 30, 150 and 250.

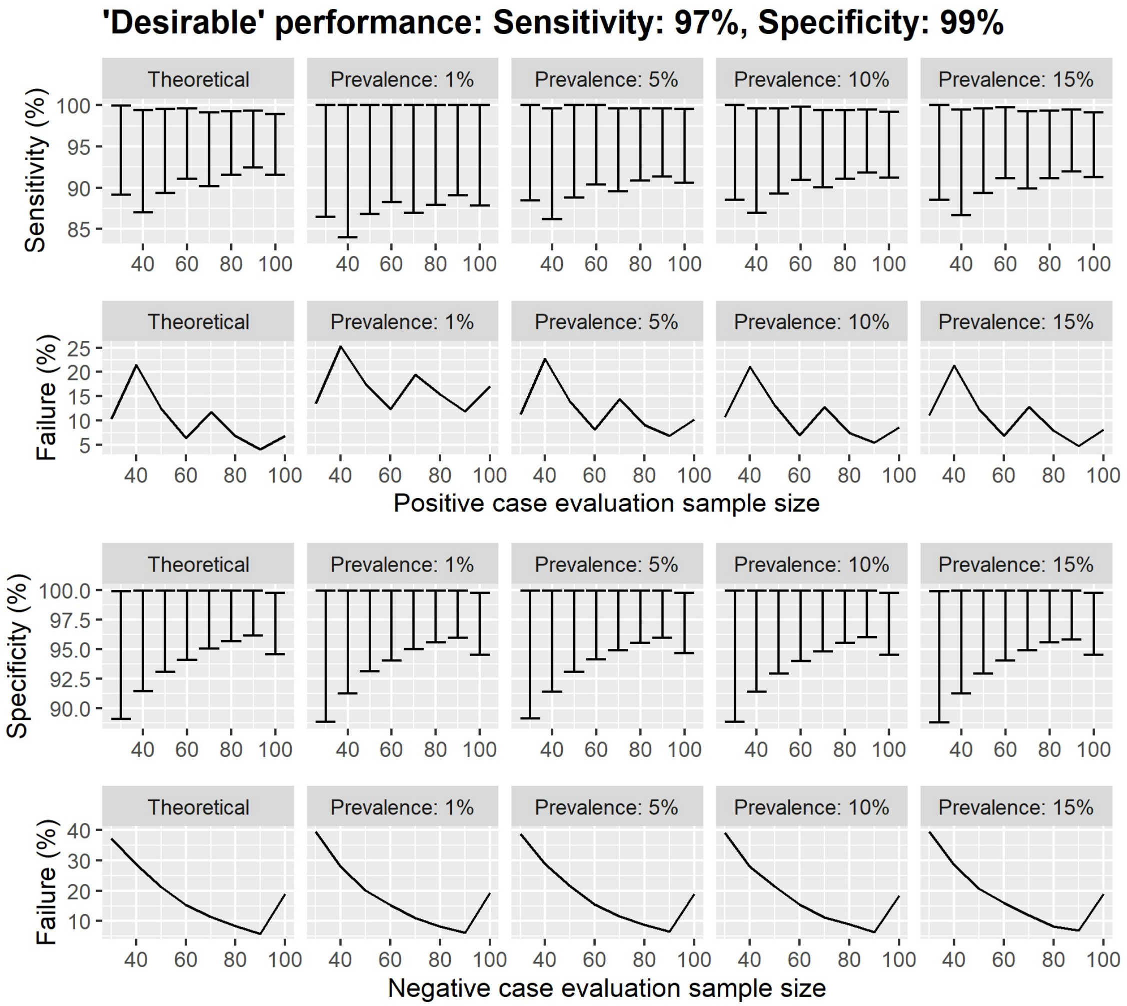

Supplementary Figure S2: D_{min} scenario estimates of real-world diagnostics accuracy from a series of Monte Carlo simulations per evaluation sample size. Each simulation consisted of 10,000 iterations each consisting of 5,000 individuals. Here the diagnostic test was assumed to achieve the minimum performance for the desirable MHRA TPP with 97% sensitivity and 99% specificity in the evaluation sample (D_{min}). The confidence intervals are displayed for sensitivity and specificity per initial evaluation sample size across different prevalence scenarios. The simulation was considered to have met the TPP confidence interval criterion if the diagnostic characteristic was above the lower 95% CI (sensitivity 93% and specificity 97%), for sample sizes between 30 and 100.

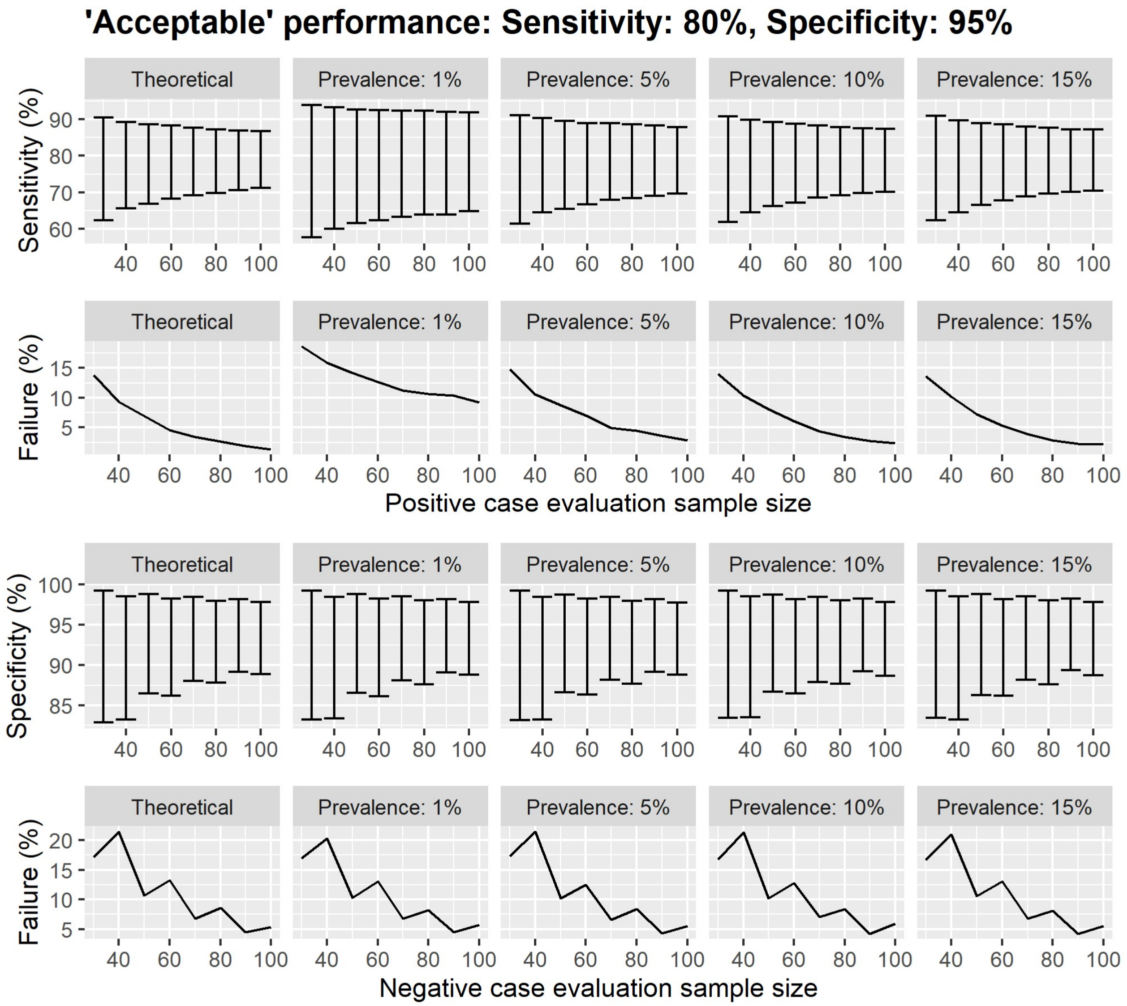

Supplementary Figure S3: A_{min} scenario estimates of real-world diagnostics accuracy from a series of Monte Carlo simulations per evaluation sample size. Each simulation consisted of 10,000 iterations each consisting of 5,000 individuals. Here the diagnostic test was assumed to achieve the minimum performance for the acceptable MHRA TPP with 80% sensitivity and 95% specificity in the evaluation sample (A_{min}). The confidence intervals are displayed for sensitivity and specificity per initial evaluation sample size across different prevalence scenarios. The simulation was considered to have met the TPP confidence interval criterion if the diagnostic characteristic was above the lower 95% CI (70% sensitivity and 90% specificity), for sample sizes between 30 and 100.

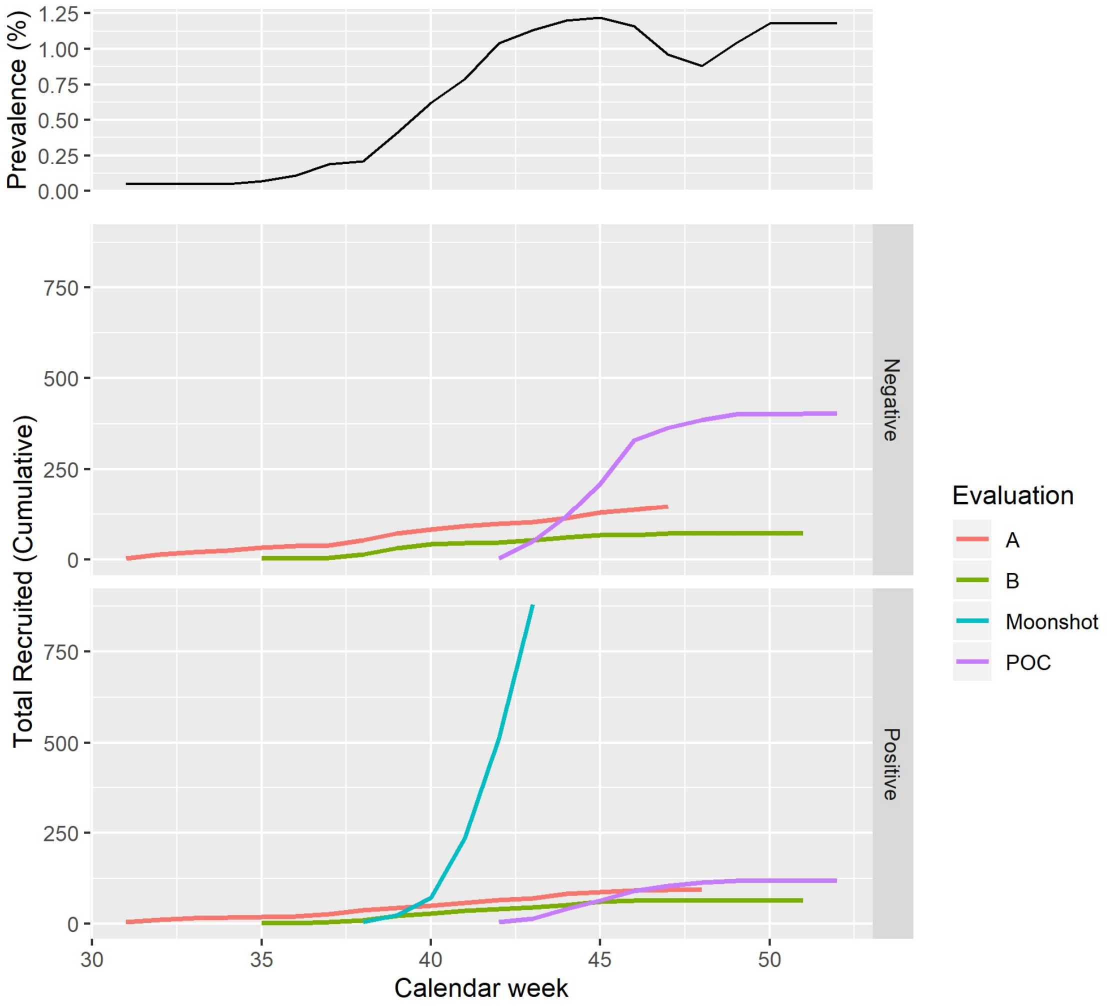

Supplementary Figure S4: Cumulative Recruitment per evaluation compared to prevalence according to the Office of National Statistics COVID-19 prevalence estimate[2]. Moonshot employed a community positive COVID-19 recall strategy based in NHS test and trace centres[1]. Point of care (POC) evaluation was a hospital based evaluation where the technology was deployed to the patient's bedside. A&B were hospital based evaluations with sample collection only, where the samples were run offsite.

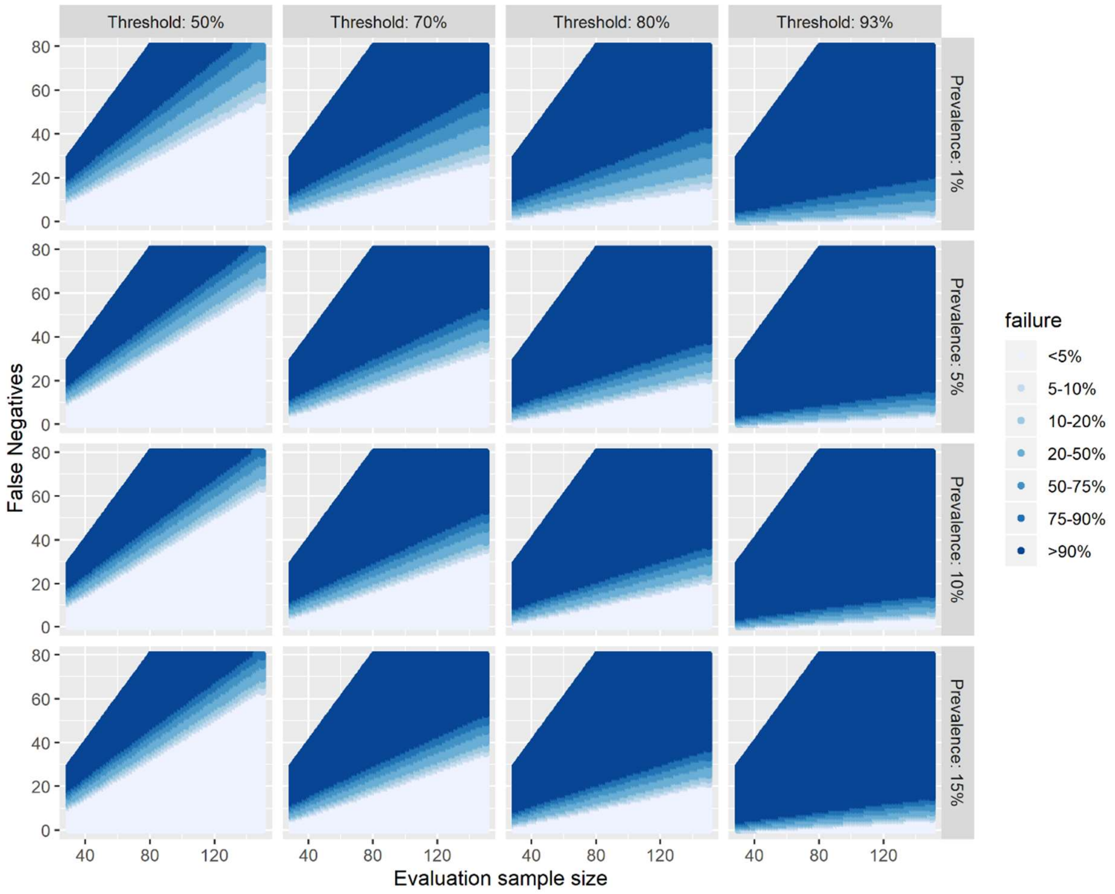

Supplementary Figure S5: Regions of the probability of failure to achieve the threshold sensitivity (thresholds: 50%, 70%, 80%, 93%) for an observed number of false negatives in a given evaluation sample size, with an assumed prevalence (prevalence: 1%, 5%, 10%, 15%) in a real-world simulation of size 5000.

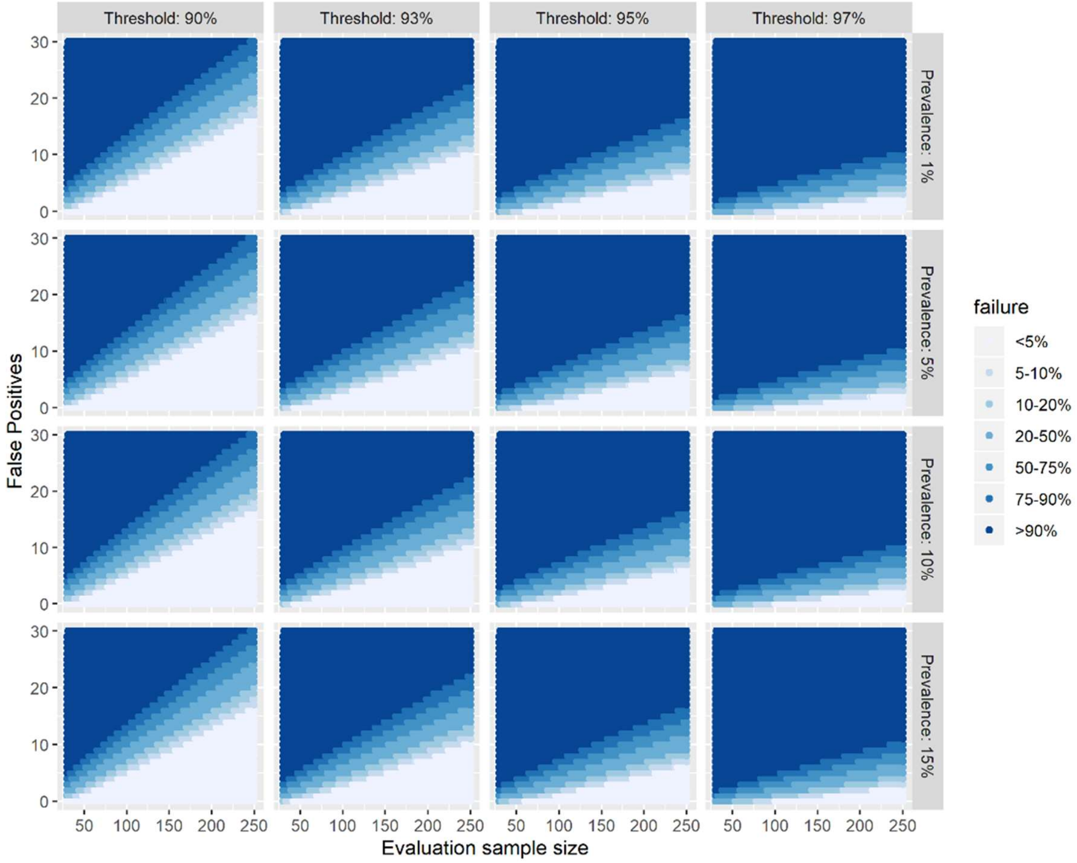

Supplementary Figure S6: Regions of the probability of failure to achieve the threshold specificity (thresholds: 90%, 93%, 95%, 97%) for an observed number of false positives in a given evaluation sample size, with an assumed prevalence (prevalence: 1%, 5%, 10%, 15%) in a real-world simulation of size 5000.

Supplementary Table S1: Estimated mean and 95% confidence intervals of the sensitivity and specificity, and the proportion that failed to meet the lower bound of the acceptable TPP criteria confidence interval (sensitivity 70%; specificity 90%) assuming the test achieved minimum performance for the acceptable TPP (A_{min}: 80% sensitivity and 95% specificity) in the evaluation sample, for a simulated population size of 5000 (Simulation Setting 2).

		Theoretical				Simulation															
						**Prevalence: 1%**				**Prevalence: 5%**				**Prevalence: 10%**				**Prevalence: 15%**			
Sensitivity																					
Evaluation sample size	Observed TP in evaluation	Mean	95% CI		Failure (%)	Mean	95% CI		Failure (%)	Mean	95% CI		Failure (%)	Mean	95% CI		Failure (%)	Mean	95% CI		Failure (%)
30	24	80.6%	65.3%	92.3%	7.7%	78.1%	57.7%	93.9%	18.6%	78.2%	61.4%	91.2%	14.8%	78.2%	61.8%	90.8%	14.0%	78.3%	62.3%	90.9%	13.6%
50	40	80.4%	68.6%	90.0%	4.0%	78.8%	61.5%	92.7%	14.1%	78.8%	65.4%	89.6%	8.8%	78.8%	66.2%	89.2%	8.1%	78.8%	66.4%	89.0%	7.3%
100	80	80.2%	71.9%	87.3%	0.9%	79.4%	64.8%	91.9%	9.2%	79.5%	69.6%	87.9%	2.9%	79.4%	70.1%	87.4%	2.4%	79.4%	70.5%	87.2%	2.1%
150	120	80.1%	73.4%	86.1%	0.2%	79.6%	66.0%	91.5%	7.6%	79.6%	71.2%	87.1%	1.3%	79.6%	71.9%	86.4%	0.8%	79.6%	72.2%	86.0%	0.6%
200	160	80.1%	74.3%	85.3%	0.1%	79.7%	66.0%	91.3%	7.2%	79.6%	71.7%	86.6%	1.0%	79.7%	72.8%	85.9%	0.4%	79.7%	73.2%	85.8%	0.2%
250	200	80.1%	74.9%	84.8%	0.0%	79.8%	66.7%	91.4%	6.1%	79.7%	72.2%	86.4%	0.6%	79.8%	73.7%	85.5%	0.1%	79.8%	73.7%	85.3%	0.1%
500	400	80.0%	76.4%	83.4%	0.0%	79.9%	67.3%	91.1%	6.0%	79.9%	73.6%	85.7%	0.2%	79.9%	74.8%	84.6%	0.0%	79.9%	75.1%	84.3%	0.0%
1000	800	80.0%	77.5%	82.4%	0.0%	79.9%	67.7%	90.7%	5.0%	79.9%	74.3%	85.2%	0.0%	79.9%	75.6%	84.1%	0.0%	79.9%	76.1%	83.5%	0.0%
2000	1600	80.0%	78.2%	81.7%	0.0%	80.0%	68.1%	90.7%	4.4%	80.0%	74.5%	85.2%	0.0%	79.9%	75.9%	83.8%	0.0%	80.0%	76.6%	83.2%	0.0%
Specificity																					
Evaluation sample size	Observed TN in evaluation	Mean	95% CI		Failure (%)	Mean	95% CI		Failure (%)	Mean	95% CI		Failure (%)	Mean	95% CI		Failure (%)	Mean	95% CI		Failure (%)
30	29	93.8%	83.3%	99.2%	17.0%	93.7%	83.2%	99.2%	17.0%	93.7%	83.2%	99.2%	17.4%	93.8%	83.5%	99.3%	16.8%	93.8%	83.5%	99.2%	16.7%
50	48	94.2%	86.5%	98.8%	10.4%	94.2%	86.6%	98.8%	10.3%	94.2%	86.6%	98.8%	10.2%	94.2%	86.7%	98.8%	10.2%	94.2%	86.3%	98.8%	10.6%
100	95	94.1%	88.8%	97.8%	5.4%	94.1%	88.8%	97.8%	5.8%	94.1%	88.8%	97.8%	5.5%	94.1%	88.7%	97.9%	6.0%	94.1%	88.8%	97.8%	5.5%
150	143	94.7%	90.7%	97.7%	1.3%	94.8%	90.7%	97.8%	1.3%	94.7%	90.6%	97.8%	1.4%	94.7%	90.6%	97.7%	1.4%	94.7%	90.6%	97.8%	1.4%
200	190	94.6%	91.0%	97.2%	0.8%	94.6%	90.9%	97.3%	0.8%	94.6%	90.9%	97.3%	0.9%	94.6%	91.1%	97.3%	0.8%	94.5%	90.9%	97.3%	0.9%
250	238	94.8%	91.8%	97.2%	0.2%	94.8%	91.7%	97.2%	0.2%	94.8%	91.7%	97.3%	0.2%	94.8%	91.7%	97.3%	0.2%	94.8%	91.7%	97.2%	0.2%
500	475	94.8%	92.7%	96.6%	0.0%	94.8%	92.6%	96.6%	0.0%	94.8%	92.6%	96.7%	0.0%	94.8%	92.6%	96.7%	0.0%	94.8%	92.6%	96.7%	0.0%
1000	950	94.9%	93.5%	96.2%	0.0%	94.9%	93.3%	96.3%	0.0%	94.9%	93.3%	96.3%	0.0%	94.9%	93.3%	96.3%	0.0%	94.9%	93.3%	96.3%	0.0%
2000	1900	95.0%	94.0%	95.9%	0.0%	95.0%	93.8%	96.0%	0.0%	95.0%	93.8%	96.0%	0.0%	95.0%	93.7%	96.1%	0.0%	95.0%	93.8%	96.1%	0.0%

Supplementary Table S2: A_{min} scenario Estimated mean and 95% confidence intervals of the sensitivity and specificity of a diagnostic test, given the test achieved 80% sensitivity and 95% specificity in the evaluation sample (A_{min}), for a simulated population size of 5000, where the evaluation sample sizes correspond to current guidelines.

Evaluation sample size	Sensitivity				Specificity			
Positive (e_+) /negative (e_-)	Mean	95% CI		Failure (%)	Mean	95% CI		Failure (%)
Theoretical								
30/30	80.6%	65.3%	92.3%	7.7%	93.8%	83.3%	99.2%	17.0%
150/250	80.1%	73.4%	86.1%	0.2%	94.8%	91.8%	97.2%	0.2%
250/1000	80.1%	74.9%	84.8%	0.0%	95.0%	94.0%	95.9%	0.0%
Prevalence: 1%								
30/30	78.1%	57.7%	93.9%	18.6%	93.7%	83.2%	99.2%	17.0%
150/250	79.6%	66.0%	91.5%	7.6%	94.8%	91.7%	97.2%	0.2%
250/1000	79.8%	66.7%	91.4%	6.1%	94.9%	93.3%	96.3%	0.0%
Prevalence: 5%								
30/30	78.2%	61.4%	91.2%	14.8%	93.7%	83.2%	99.2%	17.4%
150/250	79.6%	71.2%	87.1%	1.3%	94.8%	91.7%	97.3%	0.2%
250/1000	79.7%	72.2%	86.4%	0.6%	94.9%	93.3%	96.3%	0.0%
Prevalence: 10%								
30/30	78.2%	61.8%	90.8%	14.0%	93.8%	83.5%	99.3%	16.8%
150/250	79.6%	71.9%	86.4%	0.8%	94.8%	91.7%	97.3%	0.2%
250/1000	79.8%	73.7%	85.5%	0.1%	94.9%	93.3%	96.3%	0.0%
Prevalence: 15%								
30/30	78.3%	62.3%	90.9%	13.6%	93.8%	83.5%	99.2%	16.7%
150/250	79.6%	72.2%	86.0%	0.6%	94.8%	91.7%	97.2%	0.2%
250/1000	79.8%	73.7%	85.3%	0.1%	94.9%	93.3%	96.3%	0.0%

Supplementary Table S3: Minimum sample size required for a probability of failure (to meet the required threshold) below 5% across all simulation settings, for a simulated real-world population of 5000.

Simulation Setting	Prevalence	Sensitivity Threshold	Positive Cases	Probability of Failure	Specificity Threshold	Negative Cases	Probability of Failure
D_{min}	Theoretical	93%	90	4.2%	97%	160	4.4%
D_{min}	0.01	93%	-	-	97%	160	4.5%
D_{min}	0.05	93%	120	4.9%	97%	160	4.8%
D_{min}	0.10	93%	120	4.0%	97%	160	4.5%
D_{min}	0.15	93%	90	4.8%	97%	160	4.3%
A_{min}	Theoretical	70%	50	4.0%	90%	90	4.4%
A_{min}	0.01	70%	690	4.6%	90%	90	4.5%
A_{min}	0.05	70%	70	4.9%	90%	90	4.3%
A_{min}	0.10	70%	70	4.4%	90%	90	4.2%
A_{min}	0.15	70%	70	3.9%	90%	90	4.2%

Supplementary Table S4: Time to completion estimates for different sample sizes based on evaluations conducted within CONDOR. *Moonshot did not recruit negative cases therefore no estimate is given.

		Estimate Time to completion (days)		
	Sample Size (Positive e_+ /negative e_-)	30/30	150/250	250/1000
Evaluation	Moonshot	2 (2/-)*	8 (8/-)*	12 (12/-)*
	POC	20 (20/6)	98 (98/48)	192 (164/192)
	A	38 (38/25)	203 (190/203)	810 (316/810)
	B	55 (55/49)	408 (275/408)	1632 (458/1632)

Supplementary Table S5: Estimated probability of failure (the real-world sensitivity is <93%) given the test achieved 97% sensitivity in the evaluation sample (D_{min}), assuming a prevalence of 1% and varying the simulation and evaluation sample sizes.

Evaluation sample size	Simulation sample size				
	n=5,000	n=10,000	n=20,000	n=50,000	n=100,000
30	13.5%	12.4%	11.5%	10.8%	11.0%
50	17.7%	15.7%	14.0%	12.7%	12.0%
100	17.2%	12.8%	10.6%	7.9%	7.6%
150	10.7%	6.9%	4.4%	2.4%	2.3%
200	12.1%	7.1%	4.3%	2.4%	1.8%
250	9.6%	5.3%	2.7%	1.0%	0.5%
500	8.4%	4.0%	1.4%	0.2%	0.1%
1000	7.5%	2.8%	0.6%	0.1%	0.0%
2000	6.9%	2.3%	0.4%	0.0%	0.0%

References

[1] Preliminary report from the Joint PHE Porton Down & University of Oxford SARS-CoV-2 test development and validation cell: Rapid evaluation of Lateral Flow Viral Antigen detection devices (LFDs) for mass community testing: [Internet]. University of Oxford & Public Health England; Available from: https://www.ox.ac.uk/sites/files/oxford/media_wysiwyg/UK%20evaluation_PHE%20Porton%20Down%20%20University%20of%20Oxford_final.pdf

[2] Office for National Statistics. Coronavirus (COVID-19) Infection Survey, UK: 8 January 2021 [Internet]. Available from: https://www.ons.gov.uk/peoplepopulationandcommunity/healthandsocialcare/conditionsanddiseases/bulletins/coronaviruscovid19infectionsurveypilot/8january2021/pdf
